# Fractal regulation and incident Alzheimer’s disease in elderly individuals

**DOI:** 10.1016/j.jalz.2018.03.010

**Published:** 2018-05-04

**Authors:** Peng Li, Lei Yu, Andrew S. P. Lim, Aron S. Buchman, Frank A. J. L. Scheer, Steven A. Shea, Julie A. Schneider, David A. Bennett, Kun Hu

**Affiliations:** a Division of Sleep and Circadian Disorders, Departments of Medicine and Neurology, Brigham and Women’s Hospital, Boston, MA, USA; b Division of Sleep Medicine, Department of Medicine, Harvard Medical School, Boston, MA, USA; c Rush Alzheimer’s Disease Center, Rush University Medical Center, Chicago, IL, USA; d Division of Neurology, Department of Medicine, Sunnybrook Health Sciences Centre, University of Toronto, Toronto, ON, Canada; e Oregon Institute of Occupational Health Sciences, Oregon Health and Science University, Portland, OR, USA

**Keywords:** Longitudinal cohort study, Prediction of Alzheimer’s disease, Mild cognitive impairment, Fractal physiology, Fractal regulation

## Abstract

**Introduction:**

Healthy physiological systems exhibit fractal regulation (FR), generating similar fluctuation patterns in physiological outputs across different time scales. FR in motor activity is degraded in dementia, and the degradation correlates to cognitive decline. We tested whether degraded FR predicts Alzheimer’s dementia.

**Methods:**

FR in motor activity was assessed in 1097 nondemented older adults at baseline. Cognition was assessed annually for up to 11 years.

**Results:**

Participants with an FR metric at the 10th percentile in this cohort had a 1.8-fold Alzheimer’s disease risk (equivalent to the effect of being ~5.2 years older) and 1.3-fold risk for mild cognitive impairment (equivalent to the effect of being ~3.0 years older) than those at the 90th percentile. Consistently, degraded FR predicted faster cognitive decline. These associations were independent of physical activity, sleep fragmentation, and stability of daily activity rhythms.

**Discussion:**

FR may be a useful tool for predicting Alzheimer’s dementia.

## 1 Introduction

Outputs of many physiological systems display intrinsic self-similarity, or fractal patterns, across a wide range of temporal scales from seconds up to 24 hours, suggesting an underlying fractal regulatory mechanism [1,2]. Fractal regulation (FR) challenges the traditional theory of homeostasis as it indicates that the physiological systems do not simply settle down to a stable state [1,3–5]. Numerous studies have provided an overwhelming evidence that FR is a hallmark of healthy physiology, imparting considerable physiological advantage in terms of plasticity and adaptability (i.e., system integrity despite vastly changing conditions) [1,3–5]. For instance, FR in cardiac function is degraded with aging and under varied pathological conditions [6]; and such degradation is associated with decreased survival in patients with stroke or myocardial infarction [7–9].

Our previous cross sectional study revealed that FR in motor activity is degraded with aging and in Alzheimer’s disease (AD) [10], and in a short longitudinal study, we found that the degradation of FR in motor activity over time is associated with cognitive decline in very old adults with dementia [11]. The goal of the present study is to determine whether FR perturbation at baseline in older individuals without dementia is associated with increased risk of the development of Alzheimer’s dementia and mild cognitive impairment (MCI) and rate of cognitive decline. To achieve this goal, we analyzed the data of 1097 older adults participating in the Memory and Aging Project (MAP) at Rush AD Center [12]. Subjects have been followed up for up to 11 years at the time of analysis. FR at baseline was evaluated using wrist actigraphy continuously monitored for up to 10 days. To evaluate the cognitive function and to identify Alzheimer’s dementia and MCI development, a battery of 21 neuropsychological tests and a detailed clinical evaluation were administered each year during the baseline and follow-up assessments. We hypothesized that subjects with more degraded FR at baseline were at increased risk for incident Alzheimer’s dementia and MCI and had a faster cognitive decline.

## 2 Methods

### 2.1 Subjects

The MAP is an on-going longitudinal, community-based cohort study of aging and dementia that began in 1997 [12]. Actigraphy was added in 2005. At the time of analysis (July 07, 2016), 1836 participants were enrolled in the MAP (parent study). Among them, 348 participants died; 79 withdrew before actigraphy was added; and 54 could not be followed (e.g., moving out of the state). Of the remaining 1355 who were eligible for actigraphy assessment, 16 refused to participate, and 30 had yet to have device placement. Thus, there were 1309 subjects who had actigraphy assessment(s). As compared with the parent study, these 1309 participants had similar baseline age (79.7 ± 0.2 years old, parent study: 79.9 ± 0.2, *P >* .1), but had slightly more female subjects (76.8%, parent study: 73.7%, P = .046) and higher baseline cognitive score (0.10 ± 0.02, parent study: 0.00 ± 0.02, P < .01). Further exclusion criteria in the present study were as follows: (1) baseline dementia (103); (2) bad quality baseline actigraphy (10; see Sub-Section 2.2); and (3) no valid baseline or at least one follow-up cognitive assessment to allow the determination of incident Alzheimer’s dementia and MCI and rate of cognitive decline (99). Thus, 1097 subjects (including 855 without MCI at baseline) were included in the final analysis. Written informed consent was obtained from all participants. The study was approved by the Institutional Review Boards of Rush University Medical Center and Partners Healthcare and was performed in accordance with the ethical standards laid down in the 1964 Declaration of Helsinki and its later amendments.

### 2.2 Data collection and preprocessing

**Fig. 1.**
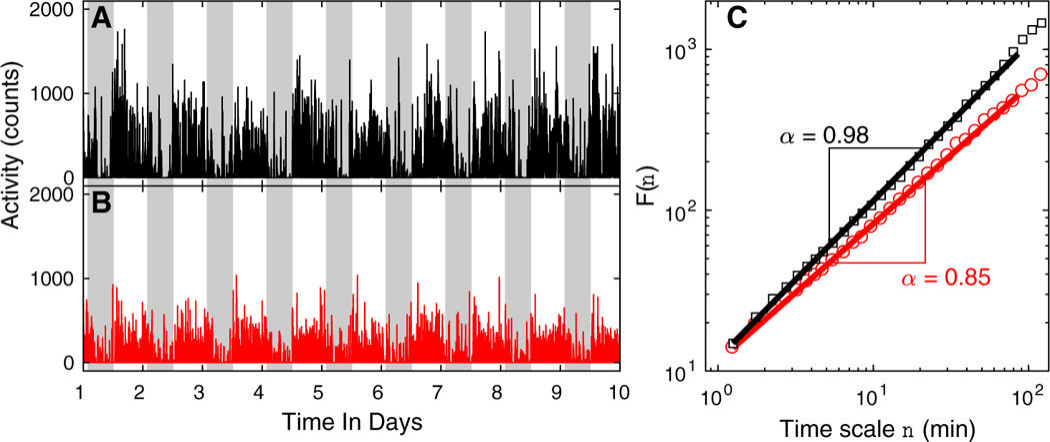
Fractal correlations in motor activity fluctuations. (A-B) Representative activity recordings of two ~ 80-year-old female subjects. Subject corresponding to (B) developed Alzheimer’s and the other corresponding to (A) did not. Gray shaded area indicates 9 PM-7 AM. (C) The corresponding detrended fluctuation analysis results of the signals in (A) and (B). The fluctuation function F(*n*) is shown versus time scale, *n*, in log-log scale, where F(*n*) is log-linearly fitted in the region of 1.25–90 min. The slope of the fitting line is defined as FR metric α. The value of α was lower for the signal in (B), indicating more random activity fluctuations. Abbreviation: FR, fractal regulation.

For assessment of daily motor activity (Fig. 1), subjects wore an activity monitor (Actical, Philips Respironics, Bend, OR) on their nondominant wrist for up to 10 days. The device predominantly measures acceleration in a direction parallel to the face of the device with a continuous sampling frequency of 32 Hz and integrates the data into a proprietary count value every 15 seconds. To ensure good signal quality, all actigraphic recordings were checked with the assistance of a self-designed MATLAB GUI program (Ver. R2015a, the MathWorks Inc., Natick, MA, USA). The most common types of quality issues were as follows: (1) isolated huge spikes with amplitude going beyond 10 standard deviations (SDs) away from the individual global mean levels; and (2) sequences of zeros with duration > 60 minutes during the daytime (likely occurred when subjects took the device off). The episodes with those issues were marked as gaps [13,14].

### 2.3 Clinical diagnoses and cognitive assessment

Cognitive function was assessed with a battery of 21 neuropsychological tests administered each year. Nineteen tests across a range of cognitive abilities were used to construct measures of five cognitive domains: (1) episodic memory (based on seven tests including Word List Recall, Word List Delay, Word List Recognition, Immediate Story Recall, Delayed Story Recall, Logical Memory Ia, and Logical Memory IIa); (2) working memory (based on three tests including Digit Span Forward, Digit Span Backward, and Digit Ordering); (3) semantic memory (based on three tests including Boston Naming, Reading Test, and Verbal Fluency); (4) perceptual speed (based on four tests including Symbol Digit, Number Comparison, Stroop Color Naming, and Stroop Word Naming); and (5) visuospatial abilities (based on two tests including Line Orientation and Progressive Matrices) [15]. To obtain domain-specific measures, individual tests within each domain were first z-scored based on the corresponding baseline means and SDs of all subjects in the cohort and then averaged across the tests. In addition, a global composite cognitive measure was obtained by averaging the 19 z-scored cognitive tests. For these cognitive measures, 0 represents the mean and 1 approximates 1 SD of the baseline score of all MAP participants. Positive scores indicate better cognitive performance.

Clinical diagnosis of Alzheimer’s dementia was based on the criteria of the joint working group of the National Institute of Neurological and Communicative Disorders and Stroke and the Alzheimer’s Disease and Related Disorders Association [16]. Diagnosis of MCI was rendered for persons who were judged to have cognitive impairment by the neuropsychologist but did not meet criteria for dementia by the clinician [17].

### 2.4 Previously proposed risk factors for AD

Several physiological functions including physical activity, sleep, and daily rhythmicity of motor activity are reportedly associated with risk of AD [18–21]. Specially, both reduced total daily activity [20] and increased sleep fragmentation [22] are associated with increased risk for incident AD in measures derived from the same actigraphic data as we use in this study. To determine the separate effects of FR on incident Alzheimer’s dementia, incident MCI, and cognitive decline, we considered and accounted for the potential effects of the following: (1) total daily activity defined by the average sum of all daily activity counts recorded [23]; (2) sleep fragmentation index developed by Lim et al. [24] that represents the probability of having an arousal (e.g., a non-zero activity count) after a long (~5 min) period of rest (i.e., sleep); and (3) interdaily stability that reflects the stability of circadian/daily activity rhythms [25].

### 2.5 Assessment of FR

To assess FR, we performed the detrended fluctuation analysis of actigraphic data to examine the temporal correlations of activity fluctuations at multiple time scales, which results in a fluctuation amplitude F(*n*) that is a function of time scales *n* [13,26]. A power-law form of F(*n*), that is, F(*n*)~*n*^α^, indicates a fractal structure in the fluctuations (Fig. 1C). The parameter α, called the scaling exponent, quantifies the temporal correlation as follows: if *α =* 0.5, there is no correlation in the fluctuations (“white noise”); if *α >* 0.5, there are positive correlations, where large values are more likely to be followed by large values (and vice versa for small values); if *α <* 0.5, there are negative correlations, where large values are more likely to be followed by small values and vice versa. α values that are close to 1.0 have been observed in many physiological outputs under healthy conditions [6,14,27,28], indicating the most complex underlying control mechanisms. We note that gaps identified in data recordings (see 2.2 Data collection and pre-processing) were skipped when performing the detrended fluctuation analysis to avoid any potential effects of interpolating missing data and/or manipulating the signal (e.g., stitching the rest data after removing the missing data) on F(*n*) [10]. Previous studies showed that cognitive impairment in patients with dementia is associated with smaller *α* at time scales < ~ 90 minutes [11]. Thus, we quantified α at time scales between 1.25 and 90 minutes in this study (Fig. 1).

### 2.6 Statistical analysis

We performed three types of statistical analyses. (1) Cox-proportional hazards models tested the association of FR metric *α* with incident Alzheimer’s dementia. The core model included baseline *α* as a predictor adjusted for demographics (i.e., age, sex, and education). To determine whether the observed association is driven by or independent of physical activity, we augmented the core model by including a term for total daily activity level (adjusted model 1). Similarly, to determine the potential effect of sleep quality, a term for sleep fragmentation index was included in the core model (adjusted model 2); and to determine the potential effect of daily activity rhythm, a term for interdaily stability was included (adjusted model 3). (2) Similar Cox-proportional hazards models (the core and three adjusted models) were used to examine the association between FR and incident MCI using a subset of 855 subjects who did not have cognitive impairment at baseline. (3) To test the association between FR metric *α* at baseline and the subsequent change in cognitive performance, a linear mixed-effect model was used with the longitudinal measure of global cognition as the dependent variable, and the time in years since baseline, baseline *α*, and the interaction between time and baseline *α* as the fixed effects. The model also included random effects for intercept and slope and was adjusted for age, sex, education, and their interactions with time. In addition, to explore whether the FR-cognition association, if exists, is domain specific, the same linear mixed-effect model was repeated for each of the five cognitive domains. These statistical analyses were performed using SAS/ STAT software (version 9.4 of the SAS System for Linux, SAS Institute Inc., Cary, NC, USA) and MATLAB Statistics and Machine Learning Toolbox (version R2016 b, The MathWorks Inc., Natick, MA, USA).

## 3 Results

### 3.1 Demographic and clinical characteristics of participants at baseline

**Fig. 2.**
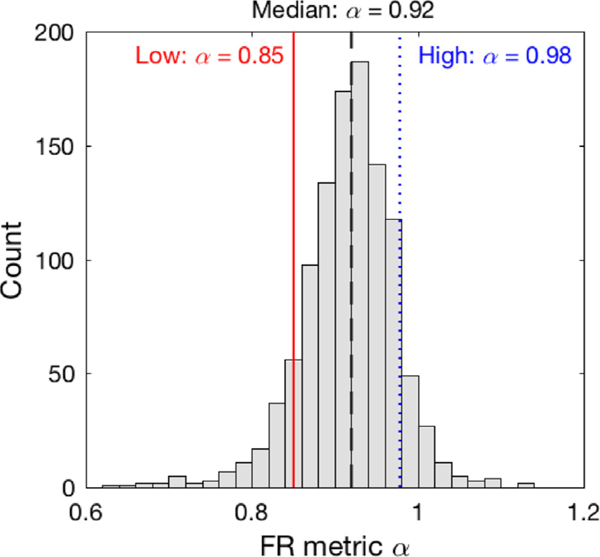
The distribution of FR metric α. The 10th percentile (“low”), median, and the 90th percentile (“high”) are highlighted in red line, black dash line, and blue dotted line, respectively. Abbreviation: FR, fractal regulation.

Demographic and clinical characteristics of participants at baseline are summarized in Table 1. Fig. 1 shows examples of baseline activity recordings and the corresponding detrended fluctuation analysis results of two subjects at the same age (~80 years old). In all 1097 participants, the FR metric a followed a normal distribution with the value ranging from ~0.6 to ~ 1.15 (Fig. 2). α values that were all >0.5 suggest positive correlations in the temporal fluctuations of motor activity in all participants. *α* showed a weak, negative association with age (Pearson *r =* −0.062, *P =* .040), that is, activity fluctuations are more random at older ages. There was a modest positive correlation between α and years of education that was marginally significant (*r* = 0.051, *P* = .092). No sex difference was found in *α* (*P* > .1). Total daily activity was positively correlated to *α* (r = 0.414, *P <* .0001). No correlation between sleep fragmentation index and α was observed (P > .3). A positive correlation between interdaily stability and *α* was observed (r = 0.186, *P* < .0001).

### 3.2 FR and incident AD dementia

**Fig. 3.**
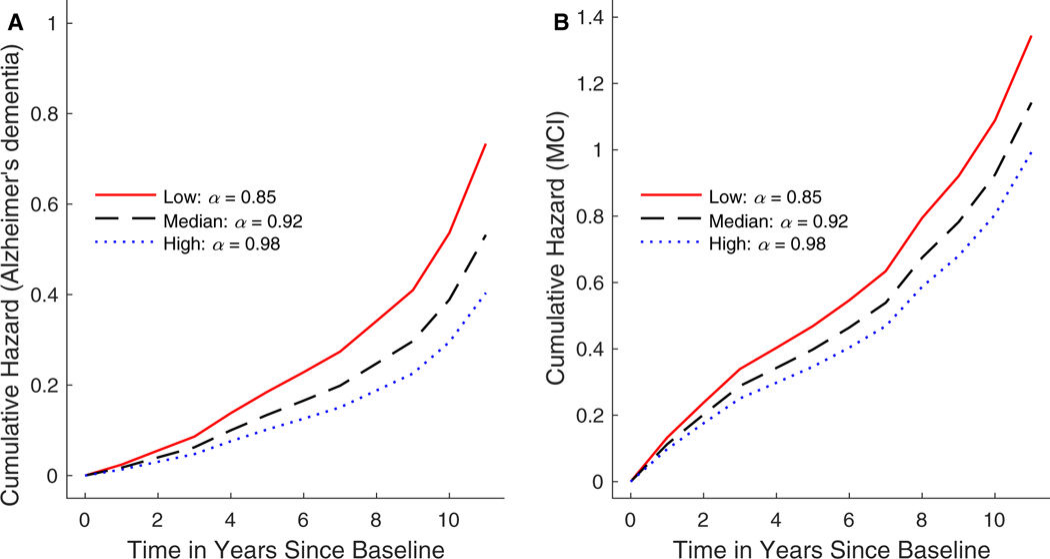
Predicted cumulative hazards of developing Alzheimer’s dementia and MCI. Panel (A) shows the risk of Alzheimer’s dementia for three representative participants with same age (81 years old), sex (females), and education (15 years), but with high (the 90th percentile at α = 0.98), median (α = 0.92), and low (the 10th percentile at *α* = 0.85) *α* levels. Panel (B) shows the risk of MCI for the same three illustrative participants. Abbreviation: MCI, mild cognitive impairment.

Over a period of up to 11 follow-up years, 220 out of 1097 (20.0%) subjects developed Alzheimer’s dementia an average of 4.6 years (median: 4; SD: 2.8) after baseline. The mean age at AD diagnosis was 89.3 (SD: 6.3) years. Using the Cox model adjusted for age, sex, and education, we found a strong association between *α* and incident Alzheimer’s dementia, with a hazard ratio (HR) of 1.31 (95% confidence interval [CI]: 1.15–1.49, *P <* .0001) for 1-SD decrease in *α* (~0.06). To illustrate the association, the predicted cumulative hazards for incident Alzheimer’s dementia by different level of *α* are shown in Fig. 3A. An individual with an *α* level at the 10th percentile (i.e., *α =* 0.85) has a 1.8-fold higher risk of developing Alzheimer’s dementia compared with an individual with an *α* level at the 90^th^ percentile (i.e., *α* = 0.98). The magnitude of the effect is equivalent to the effect of being ~5.2 years older (HR for 1 year older = 1.12; see Table 2). Indeed, the subject who had the lower FR metric *α* in Fig. 1C developed Alzheimer’s dementia 1 year after baseline; and the subject who had the higher *α* in Fig. 1C did not develop Alzheimer’s dementia over the follow-up of 6 years. The FR-Alzheimer’s dementia association remained when we included only the 855 participants who had no MCI at baseline (i.e., for 1-SD decrease in α, HR = 1.28, 95% CI 1.07–1.52, *P =* .0055).

The association between *α* and the risk of developing Alzheimer’s dementia persisted after controlling for total daily activity, sleep fragmentation, or interdaily stability (Table 2). In the model that includes both total daily activity and *α*, decreased total daily activity is also associated with an increased risk of developing Alzheimer’s dementia (for 1-SD decrease in total daily activity, HR = 1.24, 95% CI 1.03–1.49, *P =* .02), suggesting an independent effect of physical activity. In the model that includes both sleep fragmentation index and *α*, the effect of sleep fragmentation index on incident Alzheimer’s dementia was not significant (P > .1). In the model that includes both interdaily stability and *α*, the effect of interdaily stability was not significant (P > .1).

### 3.3 FR and incident MCI

Among the 855 subjects without MCI at baseline, 344 (40.2%) subjects developed MCI during the follow-up. The mean time lag was 3.4 years (median: 2; SD: 2.7) after baseline. The mean age at MCI diagnosis was 86.3 (SD: 6.1) years. After adjusting for the effects of age, sex, and education, there was an association between *α* and the risk of developing MCI, with an HR of 1.15 (95% CI: 1.02–1.29, *P =* .018) per 1-SDdecrease in α (~0.06). To illustrate the association, the predicted cumulative hazards for incident MCI by different level of α are shown in Fig. 3B. An individual with the lower α level (10th percentile) is at a 1.3-fold higher risk of developing MCI compared with an individual with the higher *α* level (90th percentile). The magnitude of the effect is equivalent to the effect of being ~ 3.0 years older.

The association between *α* and the risk of developing MCI persisted after controlling for sleep fragmentation or interdaily stability, that is, HR for 1-SD decrease in a was 1.16 after controlling for sleep fragmentation index (95% CI 1.03–1.30, *P* = .013)and was 1.16 after controlling for interdaily stability (95% CI 1.03–1.31, *P* = .015) (Table 3).When including both *α* and total daily activity in the model, the significance level for the association between *α* and the MCI risk was on borderline (*P* = .083) while the association between total daily activity and the MCI risk was not significant (*P* > .3).

### 3.4 FR and cognitive decline

**Fig. 4.**
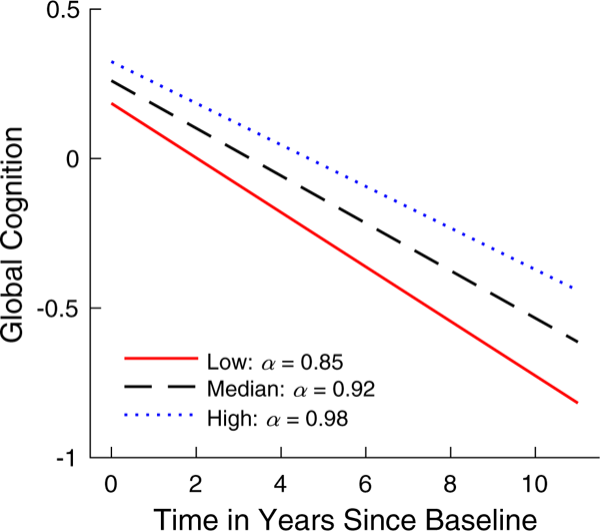
Predicted trajectory of global cognitive score. Shown the model-derived global cognitive decline for three illustrative participants with low α (the 10th percentile), median α, and high α (the 90th percentile).

Global cognition declined over time with the average annual decrease of 0.080 ± 0.004 (standard error) (*P* < .0001, Table 4). In a linear mixed-effect model adjusted for age, sex, and education, cognitive decline was much faster for subjects with smaller α, that is, for 1-SD decrease in *α*, the annual decrease in global cognition was accelerated by 0.010 ± 0.004 (*P* = .009), which represents 12.5% of the average annual decline rate and is equivalent to an accelerated decline for being 2 years older (Table 4). To illustrate the association between baseline *α* values and rate of global cognitive decline, the predicted trajectories of global cognitive score for different levels of *α* are shown in Fig. 4. The association persisted after controlling for total daily activity (for 1-SD decrease in α, estimate = 0.010 ± 0.004, *P =* .015), sleep fragmentation index (for 1-SD decrease in a, estimate = 0.093 ± 0.004, *P =* .016), or interdaily stability (for 1-SD decrease in α, estimate = 0.094 ± 0.004, *P =* .018) (Table 4).

Using similar mixed models, we also examined the associations of baseline α with the rate of decline in the five cognitive domains, separately (Tables 5–9). We found that a was consistently associated with episodic memory (for 1-SD decrease in α, estimate = 0.011 ± 0.005, *P =* .014), working memory (for 1-SD decrease in α, estimate = 0.0083 ± 0.004, *P =* .022), and perceptual speed (for 1-SD decrease in α, estimate = 0.012 ± 0.003,*P* = .0008). It was not associated with visuospatial abilities (*P =* .08) or semantic memory (*P* = .3).

The associations of *α* with episodic memory and perceptual speed remained after further controlling for total daily activity, sleep fragmentation index, or interdaily stability (all *P*’s < .05). The association of *α* with working memory also persisted after further controlling for total daily activity (*P* < .05) but was no longer significant after controlling for sleep fragmentation index or interdaily stability (both *P*’s = .05). Interestingly, the association of *α* with the decline in visuospatial abilities was significant after controlling for each of the three actigraphic measures, respectively (all *P*’s < .05). The associations of *α* with decline in semantic memory remained not significant in these augmented models (*P* = .09). We note that, after accounting for the effects of α, the cognitive declines in the five domains did not significantly depend on the baseline values of total daily activity, sleep fragmentation, and interdaily stability.

## 4 Discussion

By following a large cohort of older persons for up to 11 years, we found that subjects with more degraded FR in motor activity (i.e., weaker temporal activity correlations at time scales < 1.5 hours as characterized by lower *α* values) had a higher risk for the subsequent development of Alzheimer’s dementia and MCI. Consistently, lower *α* was also associated with a faster decline of global cognitive function over time. Those associations did not change across demographic lines (such as age, sex, and education) and were independent from other risk factors including physical activity, sleep, and daily activity rhythm.

The concept of FR has been applied in many fields of physiology to evaluate the health status of physiological systems and to predict the clinical outcomes in patients with diseases such as stroke and myocardial infarction [1,7,29]. This is the first large, community-based study to our knowledge, however, demonstrating the potential of this novel concept for predicting the risk for AD and MCI. In the cohort of the present study, the average time lag between baseline assessments and diagnosis of Alzheimer’s dementia is 4.6 years, suggesting that the perturbation of FR is already detectable 4.6 years before the Alzheimer’s dementia onset. Moreover, our results also show that the ability of FR to predict incident Alzheimer’s dementia and MCI risk is independent of many other AD risk factors including age, physical activity, sleep fragmentation, and stability of daily activity rhythms. After adjusting for the effects of α, none of these traditional measures was significantly associated with AD risk or MCI risk, except that (1) age was still associated with incident Alzheimer’s dementia and incident MCI, and (2) physical activity level was still associated with incident Alzheimer’s dementia. Our results indicate that FR may be another risk factor for AD, and its degradation may be integral to AD pathology.

Previous studies showed that AD and other brain pathologies affect diverse clinical phenotypes and are related to level of diverse motor constructs [30–34] and that poorer motor function (e.g., lower physical activity level, loss of muscle strength and bulk, and physical frailty) predicts MCI, AD, and cognitive decline [20,34]. These findings raise the hypothesis that motor dysfunction and cognitive impairment may share certain common pathophysiology (i.e., brain pathology). Supporting this hypothesis, the present study showed a strong association between degraded FR of motor activity and cognitive decline. Regarding the specific neuronal circuitries linking motor regulation and cognition, the neural network of the circadian control system may be one of the candidates because disrupting the circadian control acutely causes cognitive impairment [35,36], and long-term circadian disruption, as occurred to chronic shift work, is associated with accelerated brain aging [37]. Indeed, it is proposed that circadian dysfunction is one early sign of AD, preceding the onset of cognitive symptoms [38] and that it may exacerbate the progression of this disease [18,19,21,39]. More relevant to FR, our animal and human studies showed the circadian control system impacts fractal activity regulation [2]. In rats, lesioning of the suprachiasmatic nucleus—the well-established central circadian clock in mammals [40]—causes a breakdown of fractal activity patterns with different temporal structures at time scales < 2 hours and >4 hours [41]. In a human postmortem study, perturbed FR was associated with reductions in the amounts of two major circadian neurotransmitters in the suprachiasmatic nucleus (i.e., the vasopressinergic and neurotensinergic neurons) [42]. In addition, fractal activity patterns are perturbed in chronic shift workers during night shifts [43]; and certain interventions such as light treatments that are normally used to treat sleep and circadian disorders turn out to be beneficial for cognition and fractal activity regulation in patients with dementia [11]. Thus, circadian dysfunction may explain degraded fractal activity regulation and its prediction for AD risk.

On the other hand, it is important to note that FR is different from the regulation of daily/circadian rhythms [44]. Based on models of physical systems [45,46], it has been hypothesized that fractal physiological fluctuations reflect a network of elaborate regulatory processes interacting across multiple system components over a range of temporal and spatial scales [1]. Our animal study showed that the suprachiasmatic nucleus itself cannot generate fractal patterns [47], supporting the network theory of FR. Moreover, we found in this study that fractal activity regulation predicts AD and MCI, independent of the two accepted circadian/ sleep measures (i.e., sleep fragmentation and daily activity stability). Therefore, future studies are required to determine the neural circuitry for FR and the neuroanatomical/neuropathological changes underlying the altered FR observed in those individuals at risk for AD.

Despite the lack of fully mechanistic understanding of FR, a few recent studies have explored possible interventions that can improve or maintain FR. For instance, an animal study showed that maintaining a high physical activity level can help counteract the adverse effect of aging on FR [48]. In older adults in the middle-to-late stages of dementia, increased daily light exposure may diminish or even abolish the aging-induced degradation of FR [11]. Whether improving FR symptomatically using exercise and/or light interventions can help prevent or delay the onset of cognitive impairment merits further investigations.

Population aging is widespread across the world. How to monitor health status of the elderly reliably and cost efficiently is a contemporary challenge. Related to AD, one of the important tasks is to identify individuals at a higher risk for AD at an earlier stage when interventions may be more effective [49–52]. The rapid advances in the technology of wearable devices for assessment of daily activities provide new opportunities to address the challenge. In recent years, actigraphy has become frequently used to examine physical activity in clinical and research studies because the unobtrusive monitoring requires less cooperation from participants, allowing their usual daily behaviors [21,22,53]. In this study, we further demonstrated the value of an actigraphic measure based on nonlinear dynamic theory—FR—for predicting MCI and AD risk.

Though our findings strongly suggest the potential value of FR in AD research, a number of questions are yet to be answered. First, most of the participants in the present study were older than 65 years and primarily non-Latino whites. Can FR at young ages (<65 years) predict cognitive decline and incident Alzheimer’s dementia and MCI? Is the predictability of FR different in different racial and ethnic minorities? In addition, FR in motor activity can be temporarily affected by transient disruptions of sleep-wake cycles and circadian control (e.g., night shifts) [43]. How should actigraphic data be collected to avoid such transient, “masking” effect on FR? Besides, 9-hour continuous activity data are technically adequate to estimate FR at <1.5 hours [10,13,14]. Indeed, for healthy young subjects, we showed that the fractal activity measure at <90 min was stable from day to day [14]. However, whether it is stable in older subjects, especially those with dementia/AD, is not clear. Moreover, we only considered Alzheimer’s dementia. How specifically can FR alterations reflect AD neuropathology? How is FR affected by other types of neurodegenerative diseases such as Lewy bodies, Parkinson’s disease, vascular dementia, and frontotemporal dementia that all can lead to dementia? Follow-up studies are necessary to provide answers to these questions before the application of FR measures to diagnosis of preclinical AD.

## Figures and Tables

**Table 1:** Demographic and clinical characteristics of subjects at baseline

	Mean (SD) or N (%)	
	
Characteristics	Non-Alzheimer’s dementia	Non-MCI

N	1097	855
Female	844 (76.9%)	671 (78.5%)
Age (year)	81.0(7.4)	80.1 (7.2)
Education (years)	15.0 (3.0)	15.1 (3.0)
Global cognition	0.17 (0.53)	0.33 (0.42)
α	0.92 (0.06)	0.92 (0.06)
Total daily activity (X10^5^)	2.75 (1.54)	2.83 (1.55)
Sleep fragmentation	0.028 (0.007)	0.028 (0.006)
Interdaily stability	0.53 (0.12)	0.52 (0.12)

Abbreviations: MCI, mild cognitive impairment; SD, standard deviation.

**Table 2:** The fractal regulation metric *α* and incident Alzheimer’s dementia

	Core model		Adjusted model 1		Adjusted model 2		Adjusted model 3	
				
Terms	HR (95% CI)	*P* value	HR (95% CI)	*P* value	HR (95% CI)	*P* value	HR (95% CI)	*P* value

Age*	1.12 (1.10, 1.15)	<.0001	1.12(1.09, 1.14)	<.0001	1.12 (1.10, 1.15)	<.0001	1.12 (1.10, 1.15)	<.0001
Sex (Male)	1.08 (0.78, 1.51)	.64	1.04(0.75, 1.46)	.81	1.06 (0.74, 1.50)	.76	1.08 (0.76, 1.52)	.68
Years of education†	1.08 (1.02, 1.13)	.0036	1.07 (1.02, 1.13)	.0052	1.07 (1.02, 1.13)	.0064	1.07 (1.02, 1.13)	.0067
α‡	1.31 (1.15, 1.49)	<.0001	1.21 (1.04, 1.40)	.015	1.32 (1.15, 1.51)	<.0001	1.34 (1.16, 1.55)	<.0001
Total daily activity‡	-	-	1.24(1.03, 1.49)	.020	-	-	-	-
Sleep fragmentation index§	-	-	-	-	1.05 (0.93, 1.20)	.43	-	-
Inter-daily stability‡	-	-	-	-	-	-	0.96 (0.83, 1.12)	.64

Abbreviations: CI, confidence interval; HR, hazard ratio; SD, standard deviation.

* Results for per 1 year increase.

† Results for per 1 year decrease.

‡ Results for per 1-SD decrease.

§ Results for per 1-SD increase

**Table 3:** The fractal regulation metric α and incident MCI

	Core model		Adjusted model 1		Adjusted model 2		Adjusted model 3	
				
Terms	HR (95% CI)	*P* value	HR (95% CI)	*P* value	HR (95% CI) *P* value		HR (95% CI)	*P* value

Age*	1.09 (1.07, 1.11)	<.0001	1.09 (1.07, 1.11)	<.0001	1.09 (1.07,1.11)	<.0001	1.09 (1.07, 1.11)	<.0001
Sex (male)	1.07 (0.81, 1.40)	.65	1.07 (0.81, 1.41)	.64	1.07 (0.80, 1.42)	.66	1.08 (0.81, 1.43)	.61
Years of education†	1.00 (0.96, 1.04)	.84	1.00 (0.96, 1.04)	.89	1.00 (0.96, 1.04)	.86	1.00 (0.96, 1.04)	.86
α‡	1.14 (1.02, 1.28)	.018	1.11 (0.99, 1.26)	.083	1.16 (1.03, 1.30)	.013	1.16 (1.03, 1.31)	.015
Total daily activity‡	-	-	1.07 (0.94, 1.22)	.32	--	-	-	-
Sleep fragmentation index§	-	-	-	-	1.02 (0.91, 1.16)	.67	-	-
Interdaily stability‡	-	-	-	-	--	-	0.99 (0.87, 1.12)	.82

Abbreviations: CI, confidence interval; HR, hazard ratio; MCI, mild cognitive impairment; SD, standard deviation.

* Results for per 1 year increase.

† Results for per 1 year decrease.

‡ Results for per 1-SD decrease.

§ Results for per 1-SD increase.

**Table 4:** The fractal regulation metric *α* and decline of global cognitive function

	Core model	Adjusted model 1	Adjusted model 2	Adjusted model 3
				
Terms	Estimate (SE, *P*)	Estimate (SE, *P*)	Estimate (SE, *P*)	Estimate (SE, *P*)

Time	−0.080 (0.0042, <.0001)	−0.080 (0.0042, <.0001)	−0.081 (0.0043, <.0001)	−0.080 (0.0043, <.0001)
Age	−0.021 (0.0019, <.0001)	−0.019 (0.0020, <.0001)	−0.021 (0.0020, <.0001)	−0.021 (0.0020, <.0001)
Age × time	−0.0052 (0.00051, <.0001)	−0.0052 (0.00053, <.0001)	−0.0053 (0.00053, <.0001)	−0.0052 (0.00053, <.0001)
Sex (male)	−0.15 (0.033, <.0001)	−0.15 (0.033, <.0001)	−0.14(0.035, <.0001)	−0.16 (0.034, <.0001)
Sex (male) × time	0.0069 (0.0089, .44)	0.0069 (0.0089, .45)	0.015 (0.0092, .094)	0.012 (0.0091, .19)
Years of education	0.066 (0.0048, <.0001)	0.067 (0.0048, <.0001)	0.064 (0.0049, <.0001)	0.064 (0.0049, <.0001)
Years of education × time	0.0020 (0.0013, .11)	0.0020 (0.0013, .11)	0.0017 (0.0013, .20)	0.0017 (0.0013, .19)
α*	0.063 (0.014, <.0001)	0.047 (0.015, .0020)	0.060 (0.014, <.0001)	0.062 (0.015, <.0001)
α × time†	0.0097 (0.0037, .0090)	0.010 (0.0042, .015)	0.0093 (0.0039, .016)	0.0094 (0.0039, .018)
Total daily activity*		0.041 (0.016, .0095)		
Total daily activity × time†		−0.00064 (0.0043, .88)		
Sleep fragmentation index*			−0.045 (0.014, .0020)	
Sleep fragmentation index × time†			−0.0091 (0.0039, .019)	
Interdaily stability*				−0.0046 (0.015, .75)
Interdaily stability × time†				0.00083 (0.0039, .83)

Abbreviations: SD, standard deviation; SE, standard error.

* Results for per 1-SD change.

† Results for per 1-SD X 1-year change.

**Table 5:** The fractal regulation metric α and decline of episodic memory

	Core model	Adjusted model 1	Adjusted model 2	Adjusted model 3
Terms	Estimate (SE, *P*)	Estimate (SE, *P*)	Estimate (SE, *P*)	Estimate (SE, *P*)

Time	−0.077 (0.0052, <.0001)	−0.077 (0.0052, <.0001)	−0.079 (0.0053, <.0001)	−0.079 (0.0054, <.0001)
Age	−0.022 (0.0025, <.0001)	−0.021 (0.0026, <.0001)	−0.023 (0.0026, <.0001)	−0.022 (0.0026, <.0001)
Age × time	−0.0060 (0.00064, <.0001)	−0.0060 (0.00066, <.0001)	−0.0062 (0.00066, <.0001)	−0.0062 (0.00066, <.0001)
Sex (male)	−0.32 (0.044, <.0001)	−0.31 (0.044, <.0001)	−0.30 (0.046, <.0001)	−0.32 (0.045, <.0001)
Sex (male) × time	0.020 (0.011, .065)	0.020 (0.011, .069)	0.022 (0.011, .051)	0.022 (0.011, .053)
Years of education	0.058 (0.0063, <.0001)	0.059 (0.0063, <.0001)	0.057 (0.0064, <.0001)	0.057 (0.0065, <.0001)
Years of education × time	0.0016(0.0019, .31)	0.0016 (0.0016, .31)	0.0012 (0.0016, .45)	0.0013 (0.0016, .43)
α*	0.034 (0.018, .067)	0.015 (0.020, .44)	0.023 (0.019, .23)	0.027 (0.019, .16)
α × time†	0.011 (0.0046, .014)	0.012 (0.0052, .018)	0.014 (0.0048, .0038)	0.013 (0.0049, .0060)
Total daily activity*		0.045 (0.021, .028)		
Total daily activity × time†		−0.0017 (0.0053, .75)		
Sleep fragmentation index*			−0.047 (0.019, .014)	
Sleep fragmentation index × time†			−0.0015 (0.0048, .76)	
Interdaily stability*				−0.015 (0.019, .43)
Interdaily stability × time†				0.0025 (0.0048, .61)

Abbreviations: SD, standard deviation; SE, standard error.

* Results for per 1-SD change.

† Results for per 1-SD X 1-year change.

**Table 6:** The fractal regulation metric a and decline of working memory

Terms	Core model	Adjusted model 1	Adjusted model 2	Adjusted model 3
	Estimate (SE, *P*)	Estimate (SE, *P*)	Estimate (SE, *P*)	Estimate (SE, *P*)

Time	−0.052 (0.004, <.0001)	−0.052 (0.004, <.0001)	−0.051 (0.004, <.0001)	−0.051 (0.004, <.0001)
Age	−0.011 (0.003, <.0001)	−0.0099 (0.003, <.0001)	−0.012 (0.003, <.0001)	−0.011 (0.003, .0001)
Age × time	−0.0032 (0.0005, <.0001)	−0.0032 (0.0005, <.0001)	−0.0031 (0.0005, <.0001)	−0.0031 (0.0005, <.0001)
Sex (male)	−0.035 (0.05, .468)	−0.031 (0.048, .517)	−0.0026 (0.05, .96)	−0.022 (0.05, .66)
Sex (male) × time	0.0061 (0.009, .479)	0.0061 (0.0086, .476)	0.011 (0.009, .23)	0.0098 (0.009, .27)
Years of education	0.064 (0.007, <.0001)	0.0647 (0.0070, <.0001)	0.060 (0.007, <.0001)	0.060 (0.007, <.0001)
Years of education × time	0.00060 (0.001, .623)	0.00060 (0.001, .623)	0.00067 (0.001, .59)	0.00065 (0.001, .60)
α*	0.037 (0.020, .0679)	0.019 (0.022, .380)	0.044(0.021, <.037)	0.045 (0.021, <.037)
α × time†	0.0083 (0.004, .022)	0.0082 (0.0041, .044)	0.0072 (0.004, .056)	0.0073 (0.004, .056)
Total daily activity*		0.044 (0.0227, .051)		
Total daily activity × time†		0.00032 (0.004, .939)		
Sleep fragmentation index*			−0.043 (0.021, .042)	
Sleep fragmentation index × time†			−0.0027 (0.0037, .46)	
Interdaily stability*				0.0027 (0.021, .90)
Interdaily stability × time†				−0.00022 (0.0038, .95)

Abbreviations: SD, standard deviation; SE, standard error.

* Results for per 1-SD change.

† Results for per 1-SD X 1-year change.

**Table 7:** The fractal regulation metric α and decline of semantic memory

	Core model	Adjusted model 1	Adjusted model 2	Adjusted model 3
Terms	Estimate (SE, *P*)	Estimate (SE, *P*)	Estimate (SE, *P*)	Estimate (SE, *P*)

Time	−0.074 (0.0045, <.0001)	−0.072 (0.0046, <.0001)	−0.074 (0.0047, <.0001)	−0.073 (0.0047, <.0001)
Age	−0.013 (0.0023, <.0001)	−0.012 (0.0023, <.0001)	−0.013 (0.002, <.0001)	−0.013 (0.0023, <.0001)
Age × time	−0.0045 (0.0006, <.0001)	−0.0048 (0.00058, <.0001)	−0.0048 (0.00059, <.0001)	−0.0046 (0.00058, <.0001)
Sex (male)	−0.20 (0.040, <.0001)	−0.19 (0.040, <.0001)	−0.18 (0.041, <.0001)	−0.19 (0.041, <.0001)
Sex (male) × time	0.018 (0.0096, .061)	0.017 (0.0096, .077)	0.021 (0.010, .039)	0.017 (0.0099, .087)
Years of education	0.078 (0.0057, <.0001)	0.080 (0.0057, <.0001)	0.075 (0.0058, <.0001)	0.076 (0.0058, <.0001)
Years of education × time	0.0033 (0.0014, .017)	0.0032 (0.0014, .021)	0.0032 (0.0014, .028)	0.0029 (0.0014, .039)
α*	0.069 (0.017, <.0001)	0.050 (0.018, .0060)	0.067 (0.017, <.0001)	0.063 (0.018, .0003)
α × time†	0.0040 (0.0041, .32)	0.0077 (0.0045, .093)	0.0036 (0.0042, .40)	0.0056 (0.0043, .20)
Total daily activity*		0.047 (0.019, .011)		
Total daily activity × time†		−0.0079 (0.0046, .088)		
Sleep fragmentation index*			−0.043 (0.017, .011)	
Sleep fragmentation index × time†			−0.0060 (0.0043, .16)	
Interdaily stability*				0.030 (0.017, .082)
Interdaily stability × time†				−0.0093 (0.0043, .030)

Abbreviations: SD, standard deviation; SE, standard error.

* Results for per 1-SD change.

† Results for per 1-SD X 1-year change.

**Table 8:** The fractal regulation metric α and decline of perceptual speed

	Core model	Adjusted model 1	Adjusted model 2	Adjusted model 3
Terms	Estimate (SE, *P*)	Estimate (SE, *P*)	Estimate (SE, *P*)	Estimate (SE, *P*)

Time	−0.11 (0.0039, <.0001)	−0.11 (0.0039, <.0001)	−0.11 (0.0040, <.0001)	−0.11 (0.0040, <.0001)
Age	−0.034 (0.0029, <.0001)	−0.033 (0.0029, <.0001)	−0.035 (0.0029, <.0001)	−0.034 (0.0029, <.0001)
Age × time	−0.0052 (0.00048, <.0001)	−0.0052 (0.00050, <.0001)	−0.0053 (0.00049, <.0001)	−0.0052 (0.00049, <.0001)
Sex (male)	−0.18 (0.050, .00024)	−0.18 (0.050, .00029)	−0.16(0.051, .0014)	−0.20 (0.050, <.0001)
Sex (male) × time	0.0072 (0.0081, .37)	0.0070 (0.0082, .39)	0.010 (0.0084, .22)	0.0084 (0.0083, .31)
Years of education	0.063 (0.0072, <.0001)	0.063 (0.0072, <.0001)	0.059 (0.0072, <.0001)	0.059 (0.0073, <.0001)
Years of education × time	0.00032 (0.0012, .79)	0.00030 (0.0012, .80)	0.00012 (0.0012, .92)	0.00016 (0.0012, .89)
α*	0.13 (0.021, <.0001)	0.12 (0.023, <.0001)	0.13 (0.021, <.0001)	0.13 (0.022, <.0001)
α × time†	0.012 (0.0035, .00076)	0.012 (0.0039, .0015)	0.012 (0.0036, .00073)	0.012 (0.0036, .0010)
Total daily activity*		0.028 (0.023, .23)		
Total daily activity × time†		−0.0015 (0.0039, .70)		
Sleep fragmentation index*			−0.068 (0.021, .0016)	
Sleep fragmentation index × time†			−0.0065 (0.0036, .072)	
Interdaily stability*				−0.026 (0.021, .22)
Interdaily stability × time†				0.0020 (0.0036, .57)

Abbreviations: SD, standard deviation; SE, standard error.

* Results for per 1-SD change.

† Results for per 1-SD X 1-year change.

**Table 9:** The fractal regulation metric α and decline of perceptual orientation

	Core model	Adjusted model 1	Adjusted model 2	Adjusted model 3
Terms	Estimate (SE, *P*)	Estimate (SE, *P*)	Estimate (SE, *P*)	Estimate (SE, *P*)

Time	−0.036 (0.0042, <.0001)	−0.035 (0.0043, <.0001)	−0.036 (0.0043, <.0001)	−0.035 (0.0043, <.0001)
Age	−0.020 (0.0028, <.0001)	−0.019 (0.0028, <.0001)	−0.020 (0.0028, <.0001)	−0.019 (0.0029, <.0001)
Age × time	−0.0029 (0.00052, <.0001)	−0.0031 (0.00054, <.0001)	−0.0027 (0.00053, <.0001)	−0.0027 (0.00053, <.0001)
Sex (male)	0.33 (0.048, <.0001)	0.33 (0.048, <.0001)	0.36 (0.050, <.0001)	0.33 (0.049, <.0001)
Sex (male) × time	−0.0057 (0.0088, .52)	−0.0063 (0.0088, .47)	−0.0051 (0.0090, .57)	−0.0052 (0.0090, .56)
Years of education	0.098 (0.0069, <.0001)	0.098 (0.0069, <.0001)	0.094 (0.0070, <.0001)	0.095 (0.0071, <.0001)
Years of education × time	−0.00016 (0.0013, .90)	−0.00025 (0.0013, .84)	−0.00015 (.0013, 0.91)	−0.00024 (0.0013, .85)
α*	0.062 (0.020, .0022)	0.050 (0.022, 0.023)	0.056 (0.021, .0071)	0.056 (0.021, .0078)
α × time†	0.0066 (0.0038, .079)	0.0089 (0.0042, .034)	0.0091 (0.0039, .019)	0.0095 (0.0039, .015)
Total daily activity*		0.029 (0.022, .20)		
Total daily activity × time†		−0.0052 (0.0042, .22)		
Sleep fragmentation index*			−0.056 (0.021, .0077)	
Sleep fragmentation index × time†			0.00075 (0.0039, .85)	
Interdaily stability*				0.0059 (0.021, .78)
Interdaily stability × time†				−0.0030 (0.0039, .45)

Abbreviations: SD, standard deviation; SE, standard error.

* Results for per 1-SD change.

† Results for per 1-SD X 1-year change.

## RESEARCH IN CONTEXT

1. Systematic review: Literature reviews via PubMed indicate that fractal regulation (FR) is a hallmark of healthy physiology. Yet, the relationship between FR degradation and the risk for mild cognitive impairment and Alzheimer’s disease (AD) is unknown.
2. Interpretation: Using a large, longitudinal cohort of elderly subjects (mean age: 81 years), this study showed for the first time, to our knowledge, that degraded FR predicts the risk of AD, independent of age, physical activity, sleep, and stability of daily activity rhythms.
3. Future directions: Future studies are warranted to examine (1) whether FR can predict AD risk at younger ages (e.g., <65 years old); (2) what neuroanatomical/neuropathological changes perturb FR; (3) what are the sensitivity and specificity of FR alterations in predicting AD as compared with the other types of dementia; and (4) what interventions can effectively improve/maintain FR and cognition and delay AD onset.
